# The role of three-dimensional MRI in the differentiation between angular pregnancy and interstitial pregnancy

**DOI:** 10.1186/s12884-022-04470-z

**Published:** 2022-02-18

**Authors:** Feng Gao, Ming-hua Sun, Le Fu

**Affiliations:** Department of Radiology, Shanghai First Maternity and Infant Hospital, School of Medicine, Tongji University, 200092 Shanghai, China

**Keywords:** Angular pregnancy, Interstitial pregnancy, Junctional zone, Uterus cavity, Magnetic resonance imaging

## Abstract

**Background:**

In clinical practice it is an ongoing challenge to distinguish between angular pregnancy and interstitial pregnancy. With the three-dimensional (3D) magnetic resonance imaging (MRI) being increasingly used, it is worth exploring its role in differentiating angular pregnancy from interstitial pregnancy. This study aims to investigate how 3D MRI can help reveal the differences between these two special pregnancies in the early diagnosis.

**Methods:**

We reviewed and analyzed the 3D MRI images of 50 patients with interstitial pregnancy and 55 patients with angular pregnancy retrospectively. Imaging features were identified to compare these two special pregnancies, and the ROC (Receiver Operating Characteristic) analysis was conducted to assess the diagnostic performance.

**Results:**

The significant differences of the 3D MRI imaging features between interstitial pregnancy and angular pregnancy were found in the outline of uterus cavity (*p* < 0.001), involvement of junctional zone (*p* < 0.001), the signal of surroundings (*p* = 0.005), the relationship with round ligament (*p* = 0.042), and the overlying myometrial thickness (*p* = 0.041). Furthermore, the multivariate logistic regression analysis identified a series of significant indicators for angular pregnancy, including the junctional zone involvement, being-surrounded by hyper/iso-intensity on 3D images, and the asymmetric outline of uterus cavity. Combining these three imaging features, the AUC (Area under the Curve) of ROC curve was 0.87 in distinguishing interstitial pregnancy from angular pregnancy.

**Conclusions:**

This study suggests that 3D MRI can help distinguish angular pregnancy from interstitial pregnancy in clinical practice, with the advantages that conventional MRI or ultrasound does not have. Through the significant image features, 3D MRI plays an important role in improving the timing of diagnosis, avoiding unnecessary interventions, and preventing hemorrhage in clinical practice.

**Supplementary Information:**

The online version contains supplementary material available at 10.1186/s12884-022-04470-z.

## Background

Ectopic pregnancy (EP) is the most common cause of pregnancy-related mortality in the first trimester [1]. Interstitial pregnancy is a subtype of EP, with the implantation of the embryo in the intramural or interstitial portion of the fallopian tube. Although interstitial pregnancy only accounts for 1–3% of all EP, its mortality rate is as high as 2.5%, which is 7 times greater than the overall mortality rate in EP [2, 3]. The angular pregnancy is an eccentric intrauterine pregnancy in which embryonic tissue implants in the endometrium along the lateral edge of the uterus, medial to the utero-tubal junction. The embryo may develop or abort in the uterine cavity [4], which means that angular pregnancy is an obscure entity and the outcomes are variable [5]. Patients may suffer from spontaneous abortion or uterine rupture, while some women may eventually achieve successful live births through careful observational management [6]. Due to the different causes, treatments and outcomes, timely and accurate diagnosis of angular pregnancy and interstitial pregnancy is of great significance in clinical practice.

Diagnostic laparoscopy, as a traditional method to diagnose angular pregnancy or interstitial pregnancy under direct vision, is invasive [1, 5]. Ultrasound is currently the most preferred screening tool to evaluate the site of pregnancy implantation worldwide. However, the differences between interstitial pregnancy and angular pregnancy are very subtle sometimes, and the sensibility of the conventional ultrasound is too low to distinguish them [7]. Further, although the three-dimensional (3D) ultrasound is regarded as the best way to show the entire uterine cavity, it is possible to comprehensively evaluate the relation between the gestational sac (GS) position and the uterine cavity [8, 9]. Also, it is worth noting that the experience of the sonographer plays a vital role in the performance of 3D ultrasound, which may limit the use of 3D ultrasound. In recent years, magnetic resonance imaging (MRI) has shown great advantages in the diagnosis of various forms of EP [10, 11]. The role of MRI in diagnosing interstitial pregnancy has been described in a few case reports, but the MRI image analysis criteria has not been comprehensively addressed [1, 12–15]. Also, the two-dimensional T2-weighted (T2W) sequence plays a significant role in EP cases in the current clinical practice, but it requires multiple planes [10, 13]. With the fast-imaging speed and multi-directional reconstruction capabilities, the 3D T2W images can be effectively used for the reconstruction and identification of uterine anomalies [16, 17]. However, very few studies have reported the role of 3D MRI in the EP diagnosis, especially for angular and interstitial pregnancy.

In this study, our hypothesis is that 3D T2W MRI can provide additional information to distinguish between angular and interstitial pregnancy effectively. We retrospectively reviewed the 3D images of angular pregnancy and interstitial pregnancy, and further investigated MRI features of the 3D images between these two conditions. The purpose of the study is to explore the role of 3D T2W MRI in distinguishing angular pregnancy from interstitial pregnancy in the early diagnosis and management.

## Methods

### Patients

We retrospectively reviewed the medical records from January 2013 to December 2019 in our hospital. As the Picture Archiving and Communication System (PACS) was not available and patient digital information were not consistently recorded at our hospital until 2013, we therefore limited this study to 2013 through 2019. The institutional research ethics board approved this retrospective case-control study.

**Fig. 1:**
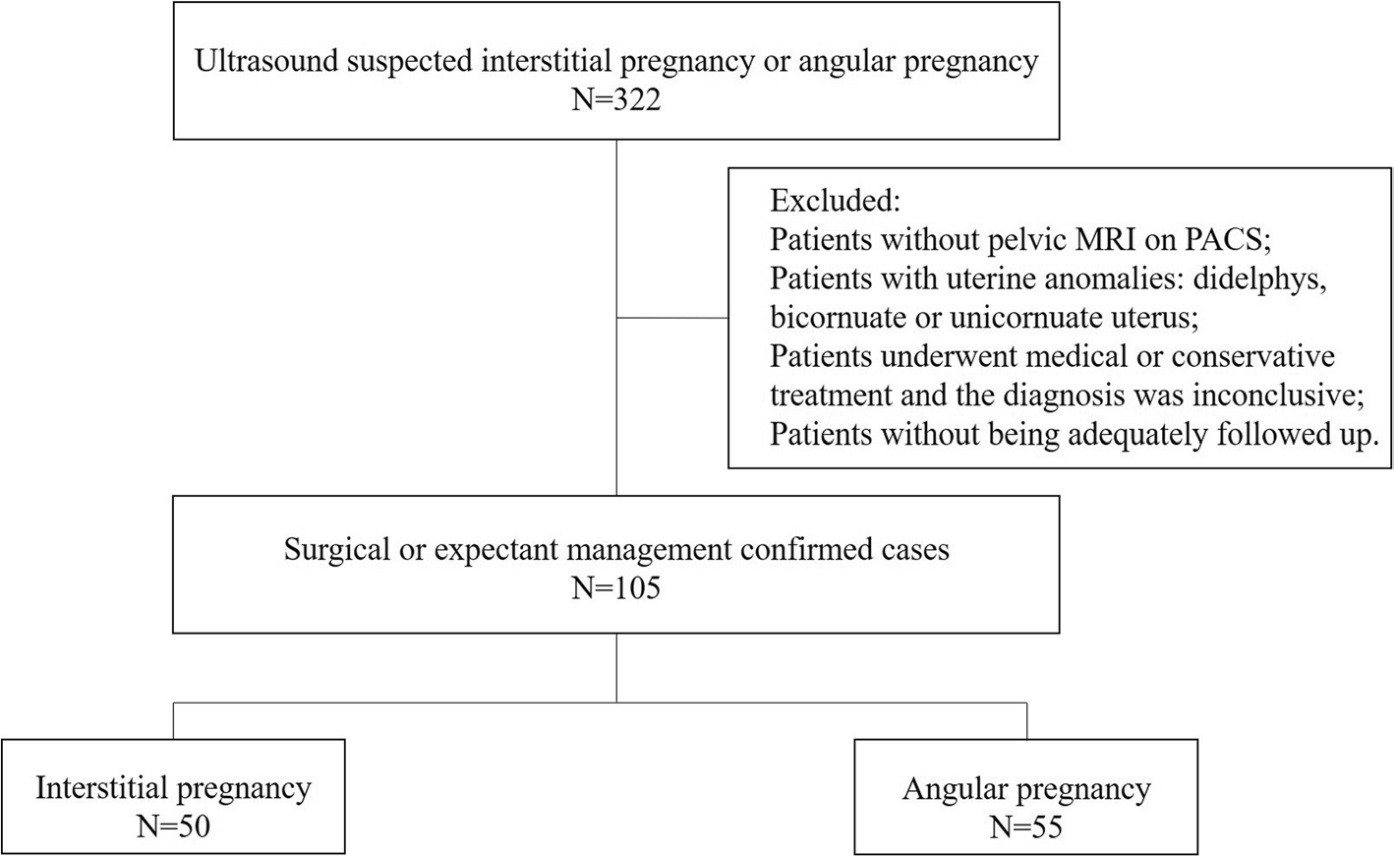
The flowchart of patients’ selection

Patients with ultrasound suspected interstitial or angular pregnancy were enrolled in the study. The flowchart of patients selection was shown in Fig. 1. The inclusion criteria were as follows: (1) patients underwent pelvic MRI and 3D T2W sequence were taken; (2) surgical or expectant management confirmed interstitial pregnancy or angular pregnancy. Also, the exclusion criteria consisted of these four as follows: (1) patients without pelvic MRI on PACS; (2) patients with uterine anomalies: didelphys, bicornuate or unicornuate uterus; (3) patients underwent medical or conservative treatment and the diagnosis was inconclusive; (4) patients without being adequately followed up. A total of 105 patients who met the above criteria were selected for this study. The case group consisted of 50 patients with surgically confirmed interstitial pregnancy Fifty-five patients were chosen as control group, and the diagnosis of them was confirmed clinically or surgically.

All the 105 patients underwent the non-contrast MRI of the pelvis. Their MRI images and clinical data were collected including patient demographics, size and morphology of the pregnancy, clinical symptoms, comorbidity, β concentration levels of the human hormone chorionic gonadotropin (β-hCG) at presentation, official ultrasound and surgical reports, and the follow-up notes.

### MRI techniques

MRI was performed on a 1.5 T unit (OPTIMA MR360, GE Medical Systems, Milwaukee, WI) using an 8-channel phased-array coil. Patients were imaged supine, with their feet entering the magnet bore first. The MRI screening sequences were as follows: axial T1-weighed fast spin-echo (FSE) imaging, axial and sagittal T2W FSE imaging with fat suppression, sagittal T2W FSE imaging without fat suppression, diffusion-weighted imaging (DWI), and coronal 3D-CUBE imaging. The protocol of the MRI sequences is summarized in Table 1. Gadolinium was not administered in any cases.

**Table 1:** The protocol of the MRI sequences

Imaging parameters	T2-weighted FSE		T1-weighted FSE	DWI(b = 800)	CubeT2-weighted
	Axial	Sagittal FSE and FRFSE	Axial	Axial	Coronal
TR(ms)/TE(ms)	3500–4000/100–130	3500–4000/100–130	400–45/10–15	4000/64	2000/91–95
section thickness(mm)	5	5	5	6	1.6
Intersection gap(mm)	1.5	1	1.5	2	0
Field of view(mm)	320	260	320	320	240
Matrix	320 × 240	288 × 192	320 × 240	128 × 128	228 × 228
Number of acquisitions	4	2	4	4	1

*TR* time of repetition, *TE* time of echo, *FSE* fast spin-echo, *FRFSE* fast recovery fast spin echo, *DWI* diffusion weighted imaging

### MRI image analysis

The MRI features were evaluated independently by two radiologists with 15 years and 6 years of experience in obstetric imaging respectively, and they both were blinded to the clinical and surgical results. Multiplanar reconstructions were performed on the Vitrea platform (GE Healthcare) using the 3D-CUBE T2W images. The MRI findings of all the 105 cases were categorized and recorded by GS-like structure size, shape, contents, signal of surroundings, involvement of junctional zone, outline of uterus cavity, relationship with the round ligament, and overlying myometrial thickness.

The GS-like structure size, shape and contents were identified according to the coronal 3D T2W images. The signal of surroundings was compared with the signal of the endometrium according to the reconstruction images. The involvement of junctional zone was identified when the low signal of the junctional zone was interrupted in any reconstruction images. Through the reconstruction of 3D T2W images, we obtained a coronal view of the uterus which provides accurate information about the shape of the cavity [18]. The outline of uterus cavity was recorded as asymmetric or regular. The round ligament was recognized on the reconstruction images and compared with the location of the GS-like structure. Also, the overlying myometrial thickness was recorded as shown on the reconstruction images.

### Statistical analysis

Pearson chi-square tests were performed to compare the differences in clinical data and MRI characteristics between the case group and control group, and Mann-Whitney U tests were performed for quantitative variables. *P* value less than 0.05 was considered statistically significant. Logistic regression analysis was performed to identify the significantly independent imaging features to differentiate angular pregnancy from interstitial pregnancy. The independent imaging features and their combinations were analyzed by Receiver operating characteristic (ROC) curves. The DeLong test was performed to evaluate the areas under the ROC curves (AUCs). Also, Kappa analysis was used to evaluate the inter-observer agreement for the MRI features. K value was interpreted as follows: poor consistency when K ≤ 0.40, moderate consistency when 0.41 ≤ K ≤ 0.60, good consistency when 0.61 ≤ K ≤ 0.80; and excellent consistency when K ≥ 0.81.

## Results

### Clinical data

In the interstitial pregnancy group, all the 50 patients underwent surgical procedures (laparotomy or laparoscopy) and the final diagnosis of interstitial pregnancy was histopathologically confirmed. Thirty-nine of these 50 patients underwent surgical management. The other 11 patients had medical and surgical management: four patients underwent surgery because β-hCG decreased slowly after single dose systemic methotrexate injection, while the local methotrexate injection was applied to 7 patients during surgery to prevent residual pregnancy conceptions. All the patients fully recovered. In the angular pregnancy group, 30 of 55 patients underwent curettage and dilatation (C&D) for the desired termination. The other 25 patients were managed expectantly to continue pregnancy. Among them, 12 patients carried to terms while 13 patients had spontaneous abortion in pregnancy.

Clinical findings between the two groups are summarized in Table 2. There were no significant differences in age, estimated gestational age and serum β-hCG level between the two groups (p>0.05). The most common clinical manifestation of the interstitial pregnancy patients was abdominal or pelvic pain (33/50, 66.0%). However, the most common clinical manifestation of the angular pregnancy patients was vaginal bleeding (40/55, 72.7%). There were six patients who had salpingectomy before in the interstitial pregnancy group, while only one patient in the angular pregnancy group. There was no significant difference in the previous salpingectomy history between the two groups (*p* = 0.068).

**Table 2:** Clinical Characteristics between two groups

	Interstitial pregnancy(*n* = 50)	angular pregnancy(*n* = 55)
age(years)	31 ± 4.65	33 ± 5.98
estimated gestational age(weeks)	7.9 ± 1.29	8.1 ± 1.31
serum β-hCG(UI/ml)	34,264 ± 1123.93	29,578.7 ± 9177.17
vaginal bleeding	27(54%)	40(73%)
abdominal or pelvic pain	33(66%)	31(56%)
previous salpingectomy	6(12%)	1(2%)

*hCG* human chorionic gonadotropin

### MRI features

**Fig. 2:**
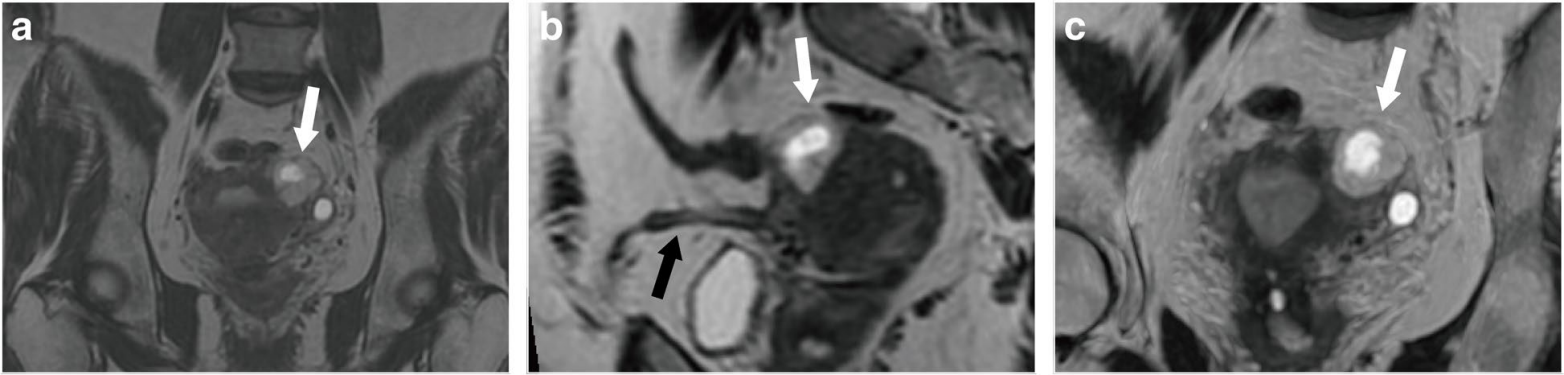
Left interstitial pregnancy in a 33-year-old woman at 6 weeks of gestation. The coronal 3D-CUBE T2WI images (**a**), reconstructed oblique sagittal image (**b**) and reconstructed oblique coronal image (**c**) revealed a cystic GS-like mass (white arrow) located lateral to the left horn of uterus, and the mass was surrounded by hypointense. Figure **b** showed the mass (white arrow) was lateral to the round ligament (black arrow). Figure **c** revealed the uterus cavity was empty and regular, and the junctional zone was intact. Laparoscopy confirmed interstitial pregnancy

**Fig. 3:**
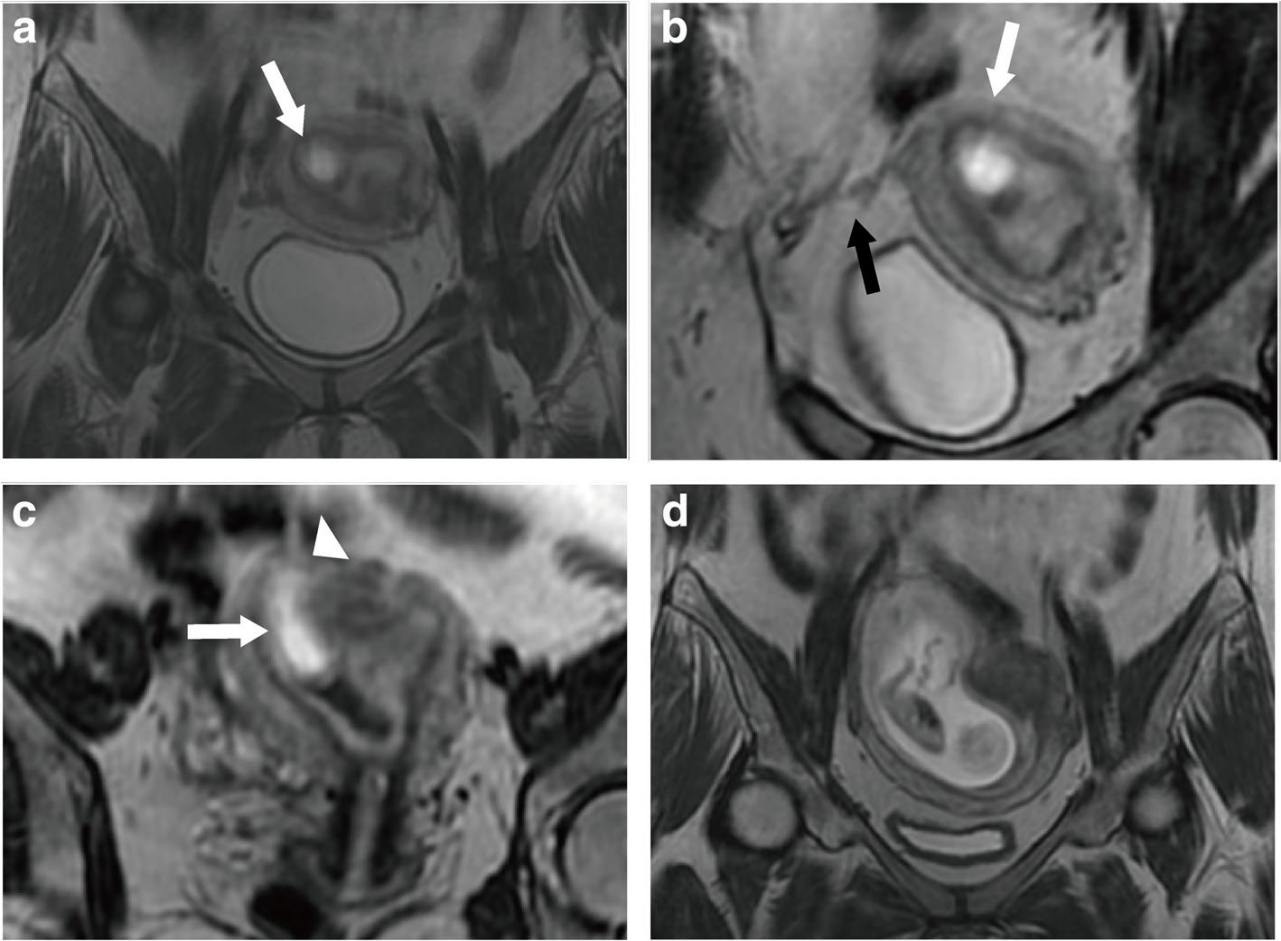
Right angular pregnancy in a 27-year-old woman at 7 weeks of gestation. The coronal 3D-CUBE T2WI images (**a**), reconstructed oblique coronal image (**b**) and reconstructed oblique axial image (**c**) revealed a cystic GS-like mass (white arrows) located at the right uterine angle and the mass was surrounded by T2 hyper/isointense endometrium. Figure **b** showed the GS was medial to the round ligament (black arrow). Figure **c** showed an interrupted junctional zone (white triangle) and the uterus cavity was asymmetric. An angular pregnancy was diagnosed according to these MRI features. The patient wanted to maintain pregnancy expectantly. After 6 weeks, this patient suffered a spontaneous abortion. The coronal Cube T2-WI(d) demonstrated the placenta was located on the right horn of the uterus and the fetus was indistinct. She underwent a C&D with ultrasound supervision

MRI findings of the two groups are summarized in Table 3. Between the two groups, significant differences were identified in the outline of uterus cavity (*p* < 0.001), the involvement of junctional zone (*p* < 0.001), the signal of surroundings (*p* = 0.005), the relationship with round ligament (*p* = 0.042), and the overlying myometrial thickness (*p* = 0.041). As shown in Fig. 2, an intact junctional zone surrounded by hypointense, lateral to the round ligament and with thinner overlying myometrial thickness, were associated with interstitial pregnancy. On the other hand, an interrupted junctional zone surrounded by hyper/iso-intensity, medial to the round ligament and with an asymmetric outline of uterus cavity, were more commonly seen in angular pregnancy (Fig. 3).

**Table 3:** Comparison of imaging features between two groups and inter-observer agreement

Imaging features	Interstitial pregnancy(N/%)	angular pregnancy(N/%)	*P*-value	*K*-value
Total number	50	55		
Size (mm)	25.14 ± 8.27	24.93 ± 8.22	0.895	0.678
Shape				
Round	20(40%)	24(44%)	0.706	0.889
Oval	30(60%)	31(56%)		
Contents				
Nonspecific liquid	15(30%)	14(25%)	0.603	0.823
Dot-like or treelike solid components	23(46%)	25(45%)	0.955	0.794
Blood	8(16%)	7(13%)	0.632	0.941
Fluid-fluid level	4(8%)	8(15%)	0.292	0.895
Surrounding T2 signal intensity^a^				0.847
Hypointensity	29(58%)	17(31%)	0.005	
Hyper, iso-intensity	21(42%)	38(69%)		
Outline of uterus cavity				0.904
Asymmetric	12(24%)	35(64%)	<0.001	
Regular	38(76%)	20(36%)		
Junctional zone				0.882
Intact	40(80%)	22(40%)	<0.001	
Interrupted	10(20%)	33(60%)		
Relationship with round ligament				
Medial	14(28%)	26(47%)	0.042	0.571
Lateral	36(72%)	29(53%)		
Overlying myometrial thickness(mm)	3.84 ± 1.52	4.44 ± 1.42	0.041	0.639

^a^ Signal intensity was compared with that of the endometrium, *GS* gestational sac

### Multivariate logistic regression analysis and the ROC curve analysis

The independent diagnostic imaging features were identified by the multivariate logistic regression analysis according to both two observers (Supplement E1 and E2), including the junctional zone involvement, being surrounded by hyper/iso-intensity, and the asymmetric outline of uterus cavity. The results demonstrate that these three imaging features are the significant indicators for angular pregnancy. The ROC curve analysis is showed in Table 4. The diagnostic model combining the three imaging features achieved the most optimal performance, better than that of any single imaging feature (*p* < 0.001 for the two observers).

**Table 4:** ROC curve analysis

	Observer 1				Observer 2			
Parameters	Sen(%)	Spe(%)	Youden index	AUC(95%)	Sen(%)	Spe(%)	Youden index	AUC(95%)
Asymmetric outline of uterus cavity	65.45	78	0.435	0.717(0.621–0.801)	65.45	80	0.455	0.727(0.632–0.810)
Hyper, iso-intensity on T2WI surrounding	69.09	64	0.331	0.665(0.567–0.755)	72.73	68	0.407	0.704(0.607–0.789)
Junctional zone involvement	60	82	0.42	0.71(0.613–0.794)	61.82	80	0.418	0.709(0.612–0.794)
Diagnostic model	74.55	80	0.546	0.849(0.766–0.911)	81.82	82	0.638	0.871(0.792–0.929)

Diagnostic model = Asymmetric outline of uterus cavity + Hyper/iso-intensity on T2WI surrounding + Junctional zone involvement

*AUC* the area under the curve, *95% CI* 95% confidence interval, *Sen* sensitivity, *Spe* specificity

### Inter-observer measurement

The inter-observer measurement for each imaging feature is indicated in Table 3. Excellent consistency between the two radiologists was found in the shape of GS, the contents of GS (except dot-like or treelike solid components), the surrounding signal intensity, the outline of uterus cavity, and the junctional zone involvement. Moderate consistency was showed in the relationship with round ligament, while good consistency was seen in the other MRI features.

## Discussion

Ultrasound is the initial imaging modality for evaluating patients with suspected interstitial pregnancy or angular pregnancy [19, 20]. Previous studies have provided the sonographic criteria for the general diagnosis of interstitial pregnancy and angular pregnancy [6, 21]. It is reported that the “interstitial line sign” has high sensitivity (80%) and specificity (98%) in the diagnosis of interstitial pregnancy [7]. Furthermore, 3D ultrasound makes it possible to delineate the uterine cavity and the intramural portion of the fallopian tube [8]. Also, 3D ultrasound is able to reveal a more confident location of the GS [22]. Edward et al. demonstrated that the niche mode of 3D ultrasound can clearly show the relation between the GS and the uterine cavity, which effectively informed the planning of the therapeutic proposal [23]. Therefore, ultrasound is essential in the diagnosis of angular pregnancy and interstitial pregnancy. However, sometimes the differences between angular pregnancy and interstitial pregnancy are very subtle. Indeed, MRI is not easily performed to investigate abnormal pregnancy due to the high cost and long queuing time for appointments. However, considering the unparalleled advantages and reliability of MRI in distinguishing between angular and interstitial ectopic pregnancies, it is of great value and significance to utilize MRI in the investigation of theses abnormal pregnancies.

MRI has served as a problem-solving procedure in ectopic pregnancy [24]. With the excellent soft tissue resolution, the large field of view and the multi-planar imaging capability, MRI has proved to be a useful tool in diagnosing ectopic pregnancies [10]. However, once the examination is finished, the conventional 2D MRI can only display limited planes. Sometimes more than three planes are required for accurate diagnosis [25], and it is difficult for technologists to determine the optimal choices of imaging planes. 3D MRI allows to construct on any desired plane after examination and does not require a precise planning of planes by the technologist. Therefore, it can add additional information to help diagnose in some cases [25, 26]. In this study, we demonstrate that 3D MRI is helpful in the differential diagnosis between the interstitial and angular pregnancy. Three significant 3D MRI imaging features were found to differentiate between these two pregnancies, including the junctional zone involvements, the surrounding signal intensity, and the outline of uterus cavity. Moreover, a final model combining these features showed a high diagnostic accuracy for both observers.

Three significant 3D MRI imaging features were included in the logistic regression model. The junctional zone is the innermost myometrium adjacent to the endometrium [27], which is best to be visualized on T2WI for evaluation and appears as a low signal region within the innermost myometrium [28]. The interruption of the junctional zone and the myometrial invasion by placental tissue suggest angular pregnancy [29]. Filhastre et al. reported two cases of interstitial pregnancy with an uninterrupted junctional zone between the gestational sac and the uterine cavity, and claim this signals interstitial pregnancy [13]. Consistent with the former study, we found that the involvement of junctional zone was a crucial imaging features to identify angular pregnancy. The junctional zone is a low signal region on the 3D T2W images, which is distinct from the high intensity signal of the endometrium and the intermediate intensity signal of the myometrium. The interruption of the junctional zone can be clearly displayed after the 3D MR image reconstruction, but sometimes this image feature is inconclusive on the orthogonal planes.

The ultrasound sign of “surrounded by endometrial” is a specific sign for angular pregnancies, with 100% specificity according to a retrospective study [19]. On the T2W image, the endometrium is usually high-intensity while the myometrium is low-intensity. Previous studies have shown that a sac surrounded by the endometrium indicates an angular pregnancy, while a sac surrounded by the myometrium indicates an interstitial pregnancy [29]. Consistent with previous studies, we demonstrate that being surrounded by hyper/iso-intensity on 3D T2WI is a hallmark for the diagnosis of angular pregnancy. However, we misdiagnosed 35 cases using this imaging feature, where all these 35 patients had a bigger size of GS-structure and more advanced gestational age. This was possibly because the decidua capsularis abuts the decidua parietalis, and the space separating them was obliterated at advanced gestational age.

The endometrial cavity is the implantation site of angular pregnancy, which could easily cause the asymmetric cavity [1]. However, the conventional ultrasound is hardly able to provide a whole view of the uterus. MRI, especially 3D imaging, allows a more accurate identification of the outline of the uterus cavity [29]. The specificity of this imaging feature reached as high as 80% in our study.

Although a myometrial mantle measurement less than 5 mm and located lateral to the round ligament have been reported as two important indicators of interstitial pregnancy [1, 30], we excluded these two image features in our diagnostic model. Similar to the results of our study, Tulandi and Al-Jaroudi argue that a thin myometrial mantle is not an important sign to distinguish the interstitial pregnancy from an eccentrically located intrauterine pregnancy [31]. We believed it is because large eccentric intrauterine pregnancy can compress the overlying myometrium and the myometrial mantle measurement can be subjective sometimes. In the angular pregnancy, the lateral uterine enlargement of the gestation displaces the round ligament reflection upward and outward, and the gestational swelling is seen medial to the round ligament [32]. However, the sensitivity of this image feature is not as high as expected in our study. We thought it is due to the bowel movement and the lack of contrast, which makes it difficult to identify the round ligament in some patients.

In addition, the use of 3D MRI could also potentially reduce the examination time. The screening time in our study was around 4 min for the 3D CUBE T2W imaging and around 2 min for each plane of the 2D T2W TSE imaging. We obtained three sequences of 2D T2W imaging for each case, so the total imaging time for the conventional 2D T2W imaging was approximately 50% longer than that of the 3D Cube T2W imaging. This result is similar to the findings of previous reports that the 3D MRI can yield time savings up to 50–60% compared with standard 2D-T2WI acquisition in three orthogonal planes [25, 26].

Although the pelvic MRI was taken in the first trimester, no adverse reactions were found in the live-born babies. Fetal cells rapidly proliferate, divide, and then undergo organogenesis in the first trimester. The implantation, migration, or differentiation of fetal cells may be disrupted due to heating theoretically. But no specific consequences of fetuses’ exposure to non-contrast MRI during the first trimester have been documented [33, 34]. Non-contrast MRI is considered safe during pregnancy by the American College of Radiology and the American College of Obstetricians and Gynecologists [35]. Andre L. Chartier’s study showed that no adverse effects regarding neonatal hearing or fetal growth were found for those who were variably exposed to 3-T MR in utero by MRI at any gestational age [36]. Considering the risk-to-benefit ratio, we suggest that the non-contrast MRI should be performed to help distinguish interstitial pregnancy from angular pregnancy.

It is acknowledged that there are some limitations in this study. First, this is a retrospective study. The inclusion criteria may result in a selection bias. Further studies may explore a cohort study to reduce the bias. Second, the sample size is relatively small and all samples are from one single institution which may hide or amplify some clinical features. A larger number of samples are expected to be collected from multi-hospital and different models should be explored using consolidated data in future.

## Conclusion

In conclusion, our results demonstrate that 3D T2W images of MRI play an important role in distinguish angular pregnancy from interstitial pregnancy. The 3D T2W image features identified on the reconstruction images, including the junctional zone involvement, asymmetric outline of uterus cavity and being surrounded by hyper/iso-intense, can strongly suggest the angular pregnancy. The combination of the above three significant imaging features can improve the diagnostic performance. Ultimately, 3D MRI enables more accurate characterization of the pregnancies for diagnosis, which can effectively avoid unnecessary interventions, prevent hemorrhage, and help to improve the diagnosis timing and patient management.

## Supplementary Information

**Additional file 1.**

## Data Availability

The datasets generated and/or analyzed during the current study are not publicly available because they contain the patients’ personal information, but are available from the corresponding author on reasonable request.
