# Supplementary material for: The role of three-dimensional MRI in the differentiation between angular pregnancy and interstitial pregnancy
Source: BMC Pregnancy Childbirth. 2022 Feb 18;22:133. doi: 10.1186/s12884-022-04470-z (PMC8857843; doi:10.1186/s12884-022-04470-z)
Supplement: Supplementary file 1 — Additional file 1. [file 12884_2022_4470_MOESM1_ESM.docx]

Table E1 Multivariate logistic regression analysis in observer 1

|  | B | S.E, | Wals | df | p | OR | 95% C.I. |
| --- | --- | --- | --- | --- | --- | --- | --- |
| Overlying myometrial thickness | 0.204 | 0.194 | 1.104 | 1 | 0.293 | 1.226 | 0.838-1.794 |
| Outline of uterus cavity | -2.493 | 0.607 | 16.882 | 1 | 0.000 | 0.083 | 0.025-0.272 |
| Surrounding T2 signal intensity | -1.828 | 0.596 | 9.411 | 1 | 0.002 | 0.161 | 0.050-0.517 |
| Relationship with round ligament | -0.635 | 0.575 | 1.218 | 1 | 0.270 | 0.530 | 0.172-1.636 |
| Junctional zone involvement | -1.929 | 0.567 | 11.578 | 1 | 0.001 | 0.145 | 0.048-0.441 |

B=regression coefficient, S.E.=standard deviation, df=degree of freedom, OR=odds ratio, C.I.=confidence interval

Table E2 Multivariate logistic regression analysis in observer 2

|  | B | S.E, | Wals | df | p | OR | 95% C.I. |
| --- | --- | --- | --- | --- | --- | --- | --- |
| Overlying myometrial thickness | 0.384 | 0.213 | 3.254 | 1 | 0.071 | 1.468 | 0.967-2.226 |
| Outline of uterus cavity | -2.677 | 0.657 | 16.581 | 1 | 0 | 0.069 | 0.019-0.249 |
| Surrounding T2 signal intensity | -1.749 | 0.575 | 9.263 | 1 | 0.002 | 0.174 | 0.056-0.537 |
| Relationship with round ligament | -2.673 | 0.667 | 16.058 | 1 | 0 | 0.069 | 0.019-0.255 |
| Junctional zone involvement | -0.893 | 0.629 | 2.015 | 1 | 0.156 | 0.41 | 0.119-1.405 |

B=regression coefficient, S.E.=standard deviation, df=degree of freedom, OR=odds ratio, C.I.=confidence interval
